# Elucidation of the mechanisms of exercise-induced hypoalgesia and pain prolongation due to physical stress and the restriction of movement

**DOI:** 10.1016/j.ynpai.2023.100133

**Published:** 2023-05-22

**Authors:** Kenichi Tanaka, Naoko Kuzumaki, Yusuke Hamada, Yukari Suda, Tomohisa Mori, Yasuyuki Nagumo, Minoru Narita

**Affiliations:** a Department of Pharmacology, Hoshi University School of Pharmacy and Pharmaceutical Sciences, 2-4-41 Ebara, Shinagawa-ku, Tokyo 142-8501, Japan; b Division of Cancer Pathophysiology, National Cancer Center Research Institute, 5-1-1 Tsukiji, Chuo-ku, Tokyo 104-0045, Japan

**Keywords:** Exercise-induced hypoalgesia, Stress, Pain prolongation, Brain reward system, Mesolimbic dopaminergic network

## Abstract

Persistent pain signals cause brain dysfunction and can further prolong pain. In addition, the physical restriction of movement (e.g., by a cast) can cause stress and prolong pain. Recently, it has been recognized that exercise therapy including rehabilitation is effective for alleviating chronic pain. On the other hand, physical stress and the restriction of movement can prolong pain. In this review, we discuss the neural circuits involved in the control of pain prolongation and the mechanisms of exercise-induced hypoalgesia (EIH). We also discuss the importance of the mesolimbic dopaminergic network in these phenomena.

## Highlights

• Moderate exercise can activate the mesolimbic dopaminergic network.
• Activation of the brain reward circuits is critical for exercise-induced hypoalgesia.
• Moderate exercise recovers stress-induced physical and emotional disturbance.

## 1 Introduction

Chronic pain has become an important social problem, not only because it significantly reduces the patient’s quality of life and makes it difficult for them to function, but also because the economic losses are enormous. Chronic pain arises due to both psychosocial and biological factors in the development and maintenance of pain. In particular, anxiety and affective disorder in particular may play important roles in establishing chronic pain (Meints and Edwards, 2018). Recently, it has been recognized that exercise therapy including rehabilitation is effective in alleviating chronic pain (Naugle et al., 2012, Leitzelar and Koltyn, 2021). On the contrary, the physical restriction of movement may affect pain prolongation. One of the mechanisms for exercise-induced hypoalgesia (EIH) involves endogenous opioid systems: β-endorphin, which is secreted under moderate exercise, decreases sensitivity to pain (Crombie et al., 2018). In addition, the mesolimbic dopaminergic network may also be activated by moderate exercise and contributes to EIH (Wakaizumi et al., 2016, Kami et al., 2018, Kami et al., 2022). In this report, based on recent findings, we review the brain circuits that are activated by moderate exercise and are associated with EIH.

## 2 Central nerves systems pathway involved in exercise-induced hypoalgesia

As reflected by the term “runner's high”, exercise can cause short-lasting feelings of euphoria, presumably through increased endogenous opioid levels followed by the activation of dopamine receptors in humans. Therefore, it has been postulated that there are close interactions between the opioid and dopaminergic systems to cause motivational behavior based on physical activity. Acute physical activity could be a form of physiological stress that increases hypothalamic–pituitaryadrenal (HPA) axis activity (Dal-Zotto et al., 2000), and short-term, but not long-term, wheel-running increases stress and aversion-related signaling in the basolateral amygdala of rats (Grigsby et al., 2022). In contrast, long-term, but not short-term, voluntary wheel-running produces rewarding effects through activation of the mesolimbic dopaminergic system in rats as measured by the conditioned place preference paradigm (Greenwood et al., 2011). Furthermore, it has been reported that voluntary exercise can decrease drug-seeking behaviors under the most challenging conditions in rats by activating reward-related signal transduction (Carroll, 2021). Thus, long-term exercise may alter the emotional state through the activation of dopamine transmission.

Consistent with these studies, moderate exercise has been reported to activate the ventral tegmental area (VTA)-nucleus accumbens (N.Acc.) pathway and induce hypoalgesia. Kami et al. (2018) found that voluntary wheel-running (VWR) activates cholinergic neurons in the laterodorsal tegmental nucleus (LDT) and orexin neurons in the lateral hypothalamus (LH), which control dopaminergic neurons in the lateral VTA via excitatory interactions. Our studies have shown that chemogenetic inhibition of the VTA-N.Acc. pathway significantly abolished the analgesic effect observed in a mouse model of neuropathic pain under treadmill exercise (Wakaizumi et al., 2016). These findings demonstrate that activation of the mesolimbic dopaminergic network is critical for the mechanism of EIH.

In addition, exercise has a variety of effects on central sensitization in the spinal cord. It has been reported that exercise suppresses neuropathic pain by increasing the levels of anti-inflammatory cytokines such as IL-4, IL-1ra and IL-5, and decreasing the expression of some growth factors such as NGF and BDNF, thereby inhibiting the activation of glial cells in the spinal cord (Almeida et al., 2015, Bobinski et al., 2018). Decreased expression of histone deacetylase 1 in Iba1-positive microglia and the concomitant promotion of hyperacetylation of H3K9 are also involved in EIH (Kami et al., 2016a). Furthermore, exercise contributes to the improvement of dysfunction of GABAergic interneurons in the spinal dorsal horn in a neuropathic pain-like state (Kami et al., 2016b).

## 3 Exercise-related energy metabolism and pain relief

In elderly patients with chronic pain, the skeletal muscle mass in both the upper and lower limbs is reduced (Sakai et al., 2017). Thus, it is assumed that, in patients with chronic pain, skeletal muscle mass may decrease as physical activity decreases due to exercise limitations. Skeletal muscle plays a central role not only in exercise and postural control but also in energy metabolism for the entire body. Recently, mitochondrial energy metabolism has been implicated in the expression of fibromyalgia (Gerdle et al., 2020, van Tilburg et al., 2020). On the other hand, it has been revealed that exercise improves mitochondrial function in skeletal muscle (Feng et al., 2013), suggesting that a combination of moderate exercise and pharmacotherapeutics may be effective for the treatment of fibromyalgia. Moreover, AMP-activated protein kinase (AMPK), which is a metabolic sensor that regulates the expression and function of TRPA1 channels, has recently been found to contribute to the development of allodynia associated with a decreased pain threshold in diabetic db/db mice with abnormal energy metabolism (Wang et al., 2018, Wang and Dai, 2021). Therefore, maintaining skeletal muscle mass and improving energy metabolism by moderate exercise are considered to be an effective therapy for pain relief.

## 4 Autonomic activity and pain relief with exercise

Pain information is processed in various regions including the somatosensory cortex, prefrontal cortex, cingulate cortex, hypothalamus, thalamus, and brainstem. These pain-sensing regions are also called the pain matrix (Tracey and Mantyh, 2007), and are strongly associated with autonomic processes (Dubé et al., 2009, Piché et al., 2010, Rouwette et al., 2012). Thus, components of the living body interact with the autonomic nervous system and pain, and these interactions largely differ in the presence of pain. Acute pain usually leads to excitation of the sympathetic nervous system, and sympathetic excitation such as activation of the descending noradrenergic system originating from the locus coeruleus attenuates pain. However, chronic pain significantly alters the normal function of the autonomic nervous system due to repeated pain stimuli (Bingel et al., 2007, Schlereth and Birklein, 2008). In particular, hyperactivity of the sympathetic nervous system induced by chronic pain such as fibromyalgia activates nociceptive afferents, which contributes to the increasing response of widespread pain, hyperalgesia, and allodynia (Martinez-Lavin, 2012). In contrast, it has also been demonstrated that chronic pain reduces the sensitivity to pain responses (Tousignant-Laflamme et al., 2006) and autonomic responses such as decreased heart rate variability and baroreflex sensitivity and prolonged response latency of the sympathetic nervous system (Tousignant-Laflamme et al., 2006, El-Badawy and El Mikkawy, 2016, Bruehl et al., 2018). Thus, the physical and mental state of patients with chronic pain get caught in a negative spiral involving not only pain sensation but also autonomic disturbance caused by pain. In response to such autonomic disturbance in the setting of chronic pain, moderate exercise is considered to be an effective treatment in patients with chronic pain. Generally, moderate exercise shifts the autonomic balance to sympathetic dominance, and slow breathing or deep breathing after exercise further shifts the autonomic balance to parasympathetic dominance (Daniela et al., 2022), suggesting that moderate exercise is helpful in recovery of a disturbed balance of the autonomic nervous system. In addition, moderate exercise has actually been shown to be effective not only in relieving pain but also in improving mental health and physical function in patients with chronic pain (Frey and Schiltenwolf, 2022), suggesting the release of endogenous endorphins that affect the activity of the autonomic nervous system. It has been well documented that endogenous opioid, 5-HT and cannabinoid systems are involved in analgesic systems in the central nervous system, and it has been hypothesized that cannabinoid receptor 1 (CB_1_) and 5-HT_1A_ receptors are particularly related to EIH in rodents (Colpaert et al., 2006, Galdino et al., 2014).

## 5 Prolonged pain due to immobilization, restraint stress, and cast fixation

The physical restriction of movement (e.g., by a cast) has occasionally been used during therapeutic interventions. Physical restriction could be a cause of stress, and it has been shown that restraint stress affects several neurotransmitters and hormonal systems followed by physiological and psychological changes as a stress response during the process of adaptation to stress. Such stress influences pain perception in two ways: by stress**-**induced analgesia**,** and by stress-induced hyperalgesia**,** which depend on the cause of stress (Li et al., 2020). On the other hand**,** it is not unusual to recognize pain as well as stiffness that is accompanied by decrease in the range of motion after restrictions like having a cast, in the area that was restrained**.** To date, the mechanisms responsible for restriction-related pain are not entirely understood, and several animal models have been developed to understand the hypersensitivity in the perception of pain, particularly for complex regional pain syndrome (CRPS), which evokes spontaneous or regional pain without an initial trauma. More interestingly, immobilization by a cast could contribute to the development of CRPS in rodents accompanied by abnormal nociception or inflammatory and other symptoms (Guo et al., 2004). Furthermore, recent studies have shown that toll-like receptor 4 (TLR4) in the microglial cells is involved in transition from acute to chronic pain in the tibial fracture-casting model of CRPS (Huck et al., 2021).

## 6 Stress-induced hyperalgesia and pain prolongation

Corticotropin-releasing hormone (CRH) neurons in the paraventricular nucleus (PVN) of the hypothalamus are known to play a role in the stress response by activating the HPA axis (Jiang et al., 2019). Recently, CRH neurons in the PVN (PVN^CRH^) have also been reported to be involved in the development of visceral hypersensitivity (Huang et al., 2021). Thus, because pain-related brain circuits overlap with stress-related brain regions, stress stimuli are expected to induce hyperalgesia. Indeed, stress stimuli have been reported to increase sensitivity to pain (Eisenberger, 2012). Furthermore, our recent study showed that activation of PVN^CRH^ accompanied by the stress-anxiety circuit prolongs postsurgical pain (Yoshida et al., 2023). On the other hand, it has been reported that the release of CRH modulates the function of dopamine neurons in the VTA (Douma and Ronald de Kloet, 2020). It has also been reported that activated dopamine neurons in the VTA indirectly suppress the activity of PVN^CRH^ (Di et al., 2020). Thus, CRH neurons and VTA dopaminergic neurons may contribute to pain modulation through their mutual interaction. Thus, it is possible that modulation of PVN^CRH^ activity may be partially involved in EIH via the activation of dopamine neurons in the VTA by moderate exercise.

## 7 Dysfunction of the brain reward circuit under the state of depression/anxiety-related chronic pain

The relationship between pain and dopaminergic neurons has attracted considerable attention. It has been reported that chronic pain reduced pre-synaptic metabolism of dopamine in the VTA as well as in the anterior cingulate gyrus and insular cortex (Wood et al., 2007). In addition, the reactivity of the mesolimbic dopamine system has been shown to decrease in response in patients with chronic pain due to the reception of persistent pain stimuli (Loggia et al., 2014, Martikainen et al., 2015). Consistent with observations in human studies, chronic pain in rodents decreased dopamine release in the nN.Acc., and pain relief was associated with increased dopamine levels in the N.Acc. shell (Navratilova et al., 2015, Serafini et al., 2020). It has also been reported that chronic pain resulted in decreased c-Fos activity in the VTA (Narita et al., 2003, Niikura et al., 2010). Thus, there is now sufficient evidence from animal models and clinical studies to suggest that chronic pain causes emotional disturbances associated with decreased dopamine release (Taylor et al., 2016, Serafini et al., 2020). Our previous studies have also found that both the firing frequency of dopaminergic neurons projecting from the VTA to the N.Acc. and dopamine release are significantly reduced in mouse models of neuropathic pain and bone cancer pain, and that optogenetic activation of a VTA-N.Acc. pathway significantly improves hyperalgesia in these models (Watanabe et al., 2018). These findings indicated that dysfunction of the VTA-N.Acc. pathway may contribute to pain prolongation.

The N.Acc. contains two types of medium spiny neurons (MSN) that express dopamine D1 or dopamine D2 receptors, respectively, and the release of endogenous dopamine induces an increase in neuronal activity in D1-MSN and an inhibition of neuronal activity in D2-MSN. D1-MSN projects directly to GABAergic interneurons in the VTA, and activation of this D1-MSN has been reported to cause the activation of dopaminergic neurons in the VTA via the disinhibition of GABAergic interneurons (Bocklisch et al., 2013). It has been reported that firing of D2-MSNs, but not D1-MSNs, in the N.Acc. was significantly increased under a neuropathic pain-like state, and the chemogenetic inhibition of D2-MSNs in the N.Acc. could suppress neuropathic pain in mice (Ren et al., 2016). Therefore, we investigated the effects of optogenetic activation of D1-MSN or inactivation of D2-MSN on neuropathic pain, and found that hyperalgesia was significantly improved in both cases (Sato et al., 2022).

Interestingly, chronic pain is highly comorbid with depression and anxiety (Dersh et al., 2002, Mlost et al., 2019). Notably, most patients (up to 80%) with major depression or Parkinson's disease report comorbid pain states (Chaudhuri et al., 2006, Young Blood et al., 2016, Mlost et al., 2019). There is evidence that chronic pain involves hypodopaminergic conditions (Defazio et al., 2008, Jarcho et al., 2012, Borsook et al., 2016), which may reduce reward reactivity and contribute to reduced motivation and depression in patients with chronic pain (Finan and Smith, 2013, Schwartz et al., 2014). The pontine parabrachial nucleus (PBN) corresponds to taste, pain, and many visceral sensations and has been suggested to play an important role in motivation (Wu et al., 2012, Berridge and Kringelbach, 2015). The PBN, located in the pons of the brainstem, receives direct projections from secondary neurons in the dorsal horn of the spinal cord, and projects to the central nucleus of the amygdala (CeA), which is involved in fear memory of pain and escape behavior in response to pain (Han et al., 2015, Sato et al., 2015, Campos et al., 2018). It has been reported that neural activity in the PBN is also involved in dysfunction of this dopaminergic nervous system (Sun et al., 2020, Markovic et al., 2021, Yang et al., 2021). The spinal-parabrachial-mesencephalic circuit may also be involved in the pathophysiology of chronic pain (Berridge and Kringelbach, 2015). An analysis using several mouse models of pain revealed that glutamatergic neurons in the PBN (PBN^Glu^) project to the VTA and substantia nigra (SN), and that GABAergic neurons in the SN (SN^GABA^) that receive projection from the PBN^Glu^ not only directly but also indirectly suppress dopaminergic neurons in the VTA by inhibiting a PBN^Glu^-VTA pathway (Yang et al., 2021). Interestingly, inhibition of neuronal activity of PBN^Glu^-SN significantly improved the decreased pain threshold in a mouse model of neuropathic pain, suggesting that the PBN^Glu^-SN^GABA^-VTA pathway may be deeply involved in pain formation and maintenance as well as in emotional disturbance caused by chronic pain (Yang et al., 2021).

Although only limited studies are available on the dopaminergic involvement in the analgesic effects of antidepressants, the descending release of dopamine in the spinal cord plays an important role in pain modulation. Intrathecal injection of bupropion produces anti-hyperalgesic effect in neuropathic rats, consistent with increases in noradrenaline and dopamine in the spinal cord (Hoshino et al., 2015, Bravo et al., 2019). Descending pain regulation is mediated primarily by monoaminergic pathways. The balance between pain promotion and pain control mediated by these dopaminergic pathways contributes to top-down pain regulation (Benarroch, 2008, Lopez-Alvarez et al., 2018, Li et al., 2019).

## 8 Effect of an enriched environment on pain relief

A so-called enriched environment (EE) has been defined as “the provision of a stimulating and structured environment that facilitates motor, sensory, social, and/or cognitive activity in a voluntary or psychologically non-stressful manner” (Tai et al., 2018). EE has been shown to increase neurogenesis and improve motor function. Modulation of plasticity is a cornerstone of rehabilitation strategies to reduce maladaptive plasticity after spinal cord injury (Sánchez-Ventura et al., 2021), and EE is also considered to be an element of rehabilitation. In addition, since pain is affected by multiple factors such as psychological and environmental factors, it can be strongly suggested that EE alleviates pain. In fact, the combination of cognitive behavioral therapy (CBT) with exercise therapy is considered to facilitate pain relief (Bushnell et al., 2013, Vachon et al., 2013).

In a recent study, EE mimicked the effects of 2*-*arachidonoylglycerol (2-AG)*,* an endocannabinoid which can significantly reduce the duration of mechanical hypersensitivity and anxiety-like behaviors by decreasing the expression of the NR2B subunit of NMDA receptors via CB_1_ receptors in the thalamus (Jiang et al., 2022). Furthermore, adverse childhood experiences may increase susceptibility to cognitive impairment and deficits in brain plasticity later in life, whereas an EE rescued neonatal pain-induced cognitive deficits and normalized hippocampal long-term potentiation (Min et al., 2022). It has been demonstrated that exposure to an EE is sufficient to ameliorate corticosterone-induced visceral pain, such as that found in irritable bowel syndrome by reducing CeA microglia-modulated neuronal plasticity (Yuan et al., 2022). Thus, in recent years, there have been many papers on the protective role of EE against pain and prevention of pain chronification.

## 9 Conclusion

**Fig. 1:**
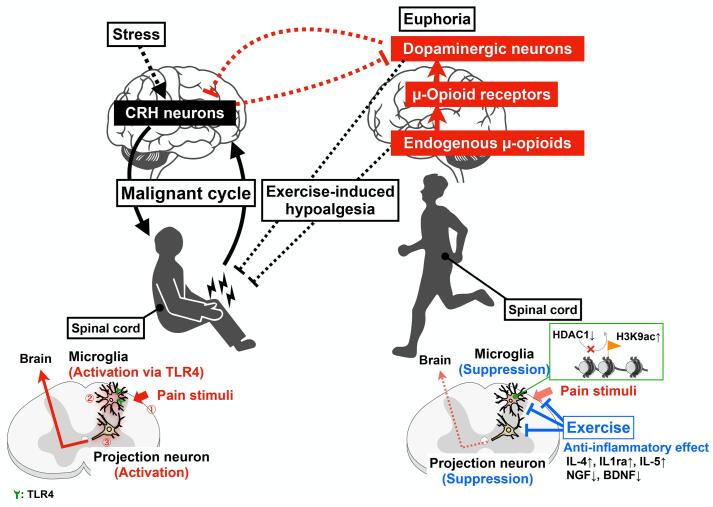
**Effects of exercise on the malignant cycle of pain and stress.** In the spinal cord, activation of microglia via TLR4 induced by cast immobilization exacerbates chronic pain (Huck et al., 2021), whereas exercise increases the production of anti-inflammatory cytokines (Almeida et al., 2015, Bobinski et al., 2018) and the level of histone acetylation in microglia (Kami et al., 2016a). In the brain, stress activates hypothalamic CRH neurons and exacerbates chronic pain (Yoshida et al., 2023). On the other hand, exercise ameliorates chronic pain through activation of the endogenous β-endorphinergic system, which is associated with the activation of dopaminergic systems (Wakaizumi et al., 2016, Kami et al., 2018, Kami et al., 2022). Furthermore, dopaminergic neurons in the ventral tegmental area could have an innervation pattern that indirectly suppresses the activity of CRH neurons in the paraventricular nucleus of the hypothalamus, suggesting that the euphoric state that arises via the activation of dopaminergic systems by exercise palliates negative emotional reactions and the exacerbation of chronic pain caused by stress.

In summary, we have outlined that moderate exercise can relieve pain by inducing activation of the mesolimbic dopaminergic network. Underlying this event is the involvement of pain-induced dysfunction of the mesolimbic dopaminergic network. On the other hand, under stress, such as under conditions that limit exercise, the firing of PVN^CRH^ triggers the prolongation of pain. In addition, PVN^CRH^ may also modulate the mesolimbic dopaminergic network (Fig. 1). These findings indicate that moderate exercise affects not only analgesia but also dopamine-related emotion, and is thus extremely important for the maintenance and improvement of physical/psychological function.

## CRediT authorship contribution statement

**Kenichi Tanaka:** Conceptualization, Writing – original draft, Writing – review & editing. **Naoko Kuzumaki:** Writing – original draft, Writing – review & editing. **Yusuke Hamada:** Writing – original draft, Writing – review & editing. **Yukari Suda:** Writing – original draft, Writing – review & editing. **Tomohisa Mori:** Writing – original draft, Writing – review & editing. **Yasuyuki Nagumo:** Writing – original draft, Writing – review & editing. **Minoru Narita:** Conceptualization, Funding acquisition, Writing – original draft, Writing – review & editing, Supervision.

## Declaration of Competing Interest

The authors declare that they have no known competing financial interests or personal relationships that could have appeared to influence the work reported in this paper.
